# Dynamic Brain State Alterations in Narcolepsy: A Hidden Markov Model Approach to Thalamocortical Instability and Symptom‐Specific Neural Correlates

**DOI:** 10.1002/brb3.71338

**Published:** 2026-03-31

**Authors:** Wenyi Li, Lijing Jia, Xingyu Wang, Yuanhui Zhao, Zhenzhong Li, Zuojun Geng

**Affiliations:** ^1^ Department of Neurology The Second Hospital of Hebei Medical University Shijiazhuang Hebei P.R. China; ^2^ The Key Laboratory of Neurology (Hebei Medical University), Ministry of Education Shijiazhuang Hebei P.R. China; ^3^ Department of Medical Imaging The Second Hospital of Hebei Medical University Shijiazhuang China

**Keywords:** Dynamic brain states, fractional occupancy, Hidden Markov Model, narcolepsy type 1, thalamocortical instability

## Abstract

**Background:**

Narcolepsy Type 1 (NT1) results from loss of hypothalamic orexin neurons, leading to unstable sleep–wake transitions. However, how this manifests as dynamic whole‐brain functional state instability remains poorly characterized.

**Methods:**

We applied Hidden Markov Modeling (HMM) to resting‐state functional magnetic resonance imaging (fMRI) data from 30 patients with NT1 and 30 age‐ and sex‐matched healthy controls to identify recurrent brain states, quantify their fractional occupancy (FO), and examine associations with clinical symptoms—specifically excessive daytime sleepiness (Epworth Sleepiness Scale, ESS) and hallucinations.

**Results:**

Five distinct dynamic brain states were identified. Compared to controls, NT1 patients showed significantly reduced FO in State 1 (thalamocortical arousal/sensory gating; *p*_FDR_ < 0.001) and elevated FO in State 4 (basal ganglia–limbic–sensorimotor integration; *p*_FDR_ < 0.001) and State 5 (reward‐introspection; *p*_FDR_ = 0.036). Notably, within the NT1 group, patients with hallucinations exhibited higher FO in State 1 than those without (*p*_FDR_ = 0.042), suggesting aberrant recruitment of this state during sleep–wake transitions. Additionally, State 4 FO showed a moderate positive correlation with ESS scores (Spearman's *ρ* = 0.38, *p*_FDR_ = 0.078).

**Conclusions:**

NT1 is associated with reduced stability of a thalamocortical alertness state and increased expression of REM‐like limbic‐subcortical network configurations. State 1 occupancy is relatively elevated in patients with hallucinations, while State 4 shows a positive association with excessive daytime sleepiness and features of REM dissociation. These findings support a dynamic, whole‐brain systems‐level framework for understanding symptom heterogeneity in NT1.

## Graphical abstract

This graphical abstract illustrates how Hidden Markov modeling of functional magnetic resonance imaging (fMRI) reveals five dynamic brain states in narcolepsy type 1 (NT1), with reduced thalamocortical arousal (State 1) and elevated REM‐like limbic‐motor (State 4) and reward‐introspection (State 5) states linking to hallucinations and daytime sleepiness, respectively.

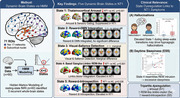

## 1 Introduction

Narcolepsy type 1 (NT1), a chronic neurological disorder characterized by excessive daytime sleepiness (EDS), cataplexy, sleep paralysis, and hypnagogic hallucinations, is pathologically associated with the selective loss of hypothalamic hypocretin/orexin neurons that disrupts sleep‐wake regulation and arousal systems (Bassetti et al. 2019). Notably, NT1 is one of the few sleep disorders with a well‐defined biological etiology, characterized by markedly reduced cerebrospinal fluid (CSF) hypocretin‐1 levels, making it an ideal human model for studying the dynamic regulation of sleep‐wake transitions.

Clinically, EDS reflects a fundamental difficulty in maintaining a stable wakeful state, whereas hypnagogic and hypnopompic hallucinations exemplify a pathological blending of sleep and wakefulness—specifically, the intrusion of dream‐like content into conscious perception during transitional states of awareness. Complementing these core symptoms, patients also exhibit selective impairments in sustained attention and executive control, while basic memory functions remain relatively preserved (Naumann et al. 2006; Witt et al. 2018). Together, this symptom‐cognition profile strongly suggests that the core deficit in NT1 is not a generalized reduction in brain function, but rather an impairment in the dynamic stability of whole‐brain functional states. This instability simultaneously undermines wakefulness maintenance (manifesting as EDS), permits abnormal intrusion of sleep‐related neural activity (leading to hallucinations), and reduces the availability of neural substrates required for higher‐order cognitive operations.

While resting‐state functional magnetic resonance imaging (rs‐fMRI) studies have identified static functional connectivity (sFC) abnormalities in narcolepsy patients, particularly within the default mode network (DMN) and salience network (SN) (Wada et al. 2019; Fulong et al. 2020), these conventional approaches fail to capture the temporal dynamics of brain activity. This limitation is particularly significant given accumulating evidence that functional connectivity exhibits dynamic temporal fluctuations, reflecting neural reorganization in response to evolving cognitive and behavioral demands (Cavanna et al. 2018).

The HMM has emerged as a powerful analytical approach for characterizing dynamic transitions of brain functional states (Stevner et al. 2019; Ezaki et al. 2021). Unlike traditional sliding‐window approaches that estimate time‐varying functional connectivity, HMM provides a model‐based framework to identify a finite set of recurring whole‐brain activity states directly from multivariate BOLD signals, without requiring arbitrary temporal windowing. Each state represents a distinct spatial pattern of regional co‐activation, and the model simultaneously estimates how frequently and for how long the brain resides in each state. This methodology has demonstrated unique advantages in studies of mild traumatic brain injury (mTBI) and major depressive disorder (MDD), revealing dynamic brain network abnormalities closely associated with cognitive dysfunction and clinical symptoms (Lu et al. 2024; Sendi et al. 2021). Given its ability to characterize transient yet structured brain configurations at high temporal resolution, HMM is particularly well‐suited for investigating narcolepsy, a disorder defined by unstable transitions between wakefulness and sleep‐related neural states.

Given that the core pathology of NT1 lies in dynamic dysregulation, this study applied HMM to resting‐state fMRI data from patients with NT1 to test whether patients exhibit reduced maintenance of a “stable alertness baseline state”—characterized by thalamocortical activation—and whether this abnormality is associated with the severity of daytime sleepiness and the frequency of hallucinations.

## 2 Materials and Methods

### 2.1 Participants

This study enrolled 35 patients with narcolepsy type 1 and 30 age‐ and sex‐matched healthy controls. Five patients were excluded due to excessive head motion during fMRI scanning (translation > 2.0 mm or rotation > 2.0°), resulting in a final sample of 30 patients. The narcolepsy group met the following criteria: (1) Diagnosis according to the International Classification of Sleep Disorders, Third Edition (ICSD‐3): Excessive daytime sleepiness (EDS) (≥ 3 months) accompanied by cataplexy (confirmed by two independent neurologists) or a CSF hypocretin‐1 level ≤ 110 pg/mL; (2) Multiple Sleep Latency Test (MSLT) demonstrating a mean sleep latency ≤ 8 min with ≥ 2 sleep‐onset REM periods (SOREMPs), or ≥ 1 SOREMP on MSLT following the observation of a nocturnal SOREMP on PSG. Healthy controls fulfilled the following: (1) Epworth Sleepiness Scale (ESS) score ≤ 10; (2) Absence of narcolepsy‐related symptoms and history of neurological/psychiatric disorders. All participants had not used medications affecting the sleep/wake cycle for ≥ 1 month prior to enrollment, and structural brain lesions were excluded by MRI. The study protocol was approved by the Institutional Ethics Committee (Approval No.: 2023‐R437), and written informed consent was obtained from all participants. Detailed demographic and clinical characteristics of the study cohort are provided in Supplementary Table S1.

### 2.2 Neuroimaging Acquisition and Processing

High‐resolution anatomical and whole‐brain functional magnetic resonance imaging (fMRI) data were acquired using a 3T GE Signa MRI scanner (General Electric Healthcare, USA) with a 64‐channel head coil. Anatomical images were obtained via a T1‐weighted three‐dimensional fast spoiled gradient‐recalled echo (3D T1W FSPGR) sequence with BRAVO technology, employing the following parameters: repetition time (TR) = 9 ms, echo time (TE) = 3.4 ms, flip angle (FA) = 15, inversion time (TI) = 450 ms, slice thickness = 1 mm, field of view (FOV) = 224 × 224 mm^2^. Functional imaging utilized a blood oxygen level‐dependent (BOLD) contrast‐sensitive gradient‐echo echo‐planar imaging (GRE‐EPI) sequence with the following specifications: TR = 2000 ms, TE = 30 ms, FA = 60, slice thickness = 3.5 mm (36 interleaved slices for whole‐brain coverage), FOV = 224 × 224 mm^2^, scan duration = 8 min, and 240 volumes. Participants with head motion exceeding 2.0 mm in translation or 2.0° in rotation were excluded from further analysis. Resting‐state fMRI (rs‐fMRI) data preprocessing was performed using the DPABI toolkit (v6.1, http://rfmri.org/dpabi) following standardized pipelines.

**FIGURE 1:**
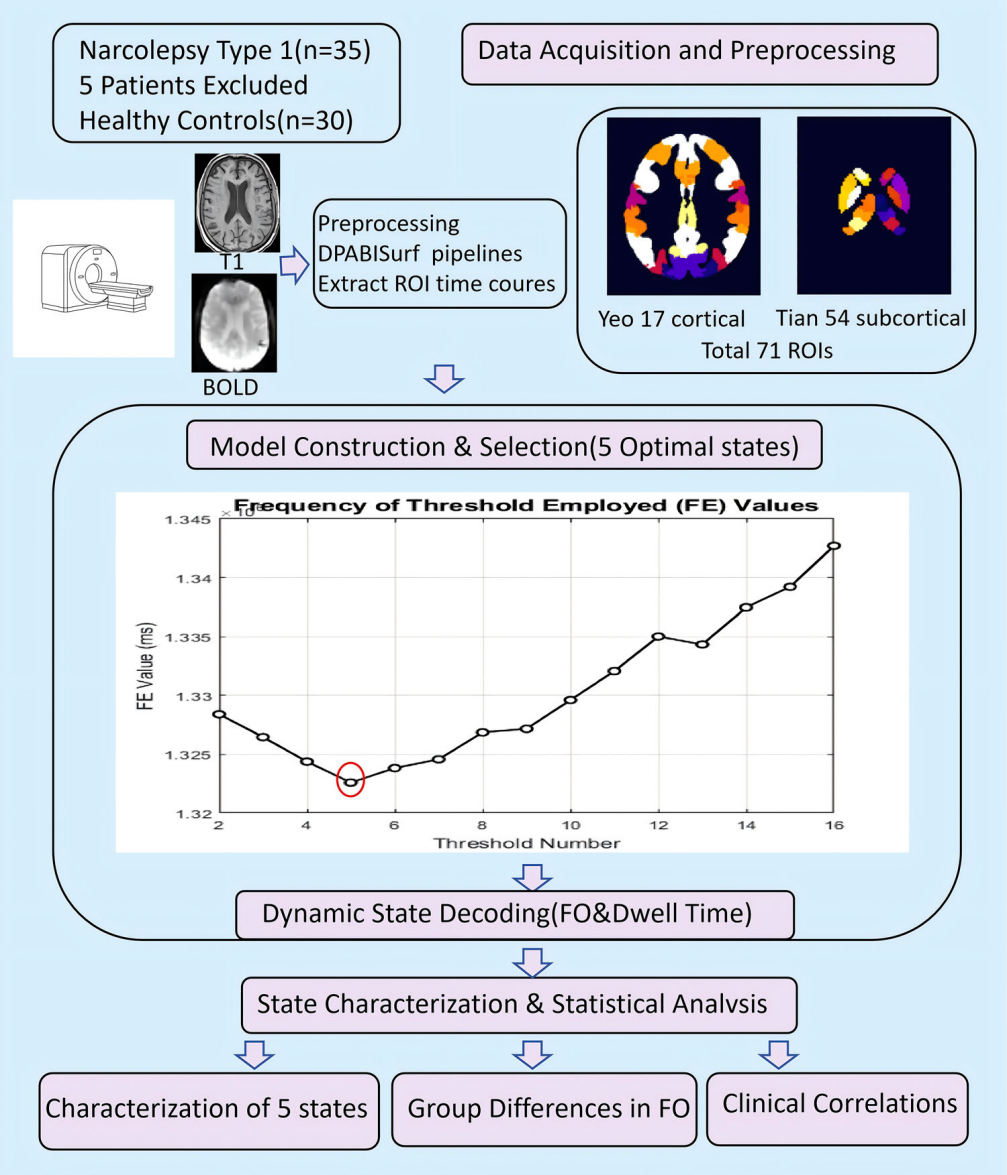
**Methodological workflow of the HMM‐based dynamic brain analysis. Data Acquisition and Preprocessing**: High‐resolution T1‐weighted anatomical and resting‐state fMRI data from 30 NT1 patients and 30 healthy controls (HC) were acquired and preprocessed via the DPABISurf pipeline. ROI Extraction: A dual‐template approach, combining the Yeo 17‐network cortical parcellation and the Tian subcortical atlas (54 regions), was used to extract time series from 71 ROIs. **HMM Model Selection**: Hidden Markov Models (HMM) were evaluated for *K* = 2 to 16 states. Free Energy (FE) minimization identified *K* = 5 (red circle) as the optimal number of brain states. **Statistical Analysis**: The five‐state configuration was further analyzed for state‐specific characterization, group differences in Fractional Occupancy (FO), and clinical correlations (ESS scores and hallucinations).

**FIGURE 2:**
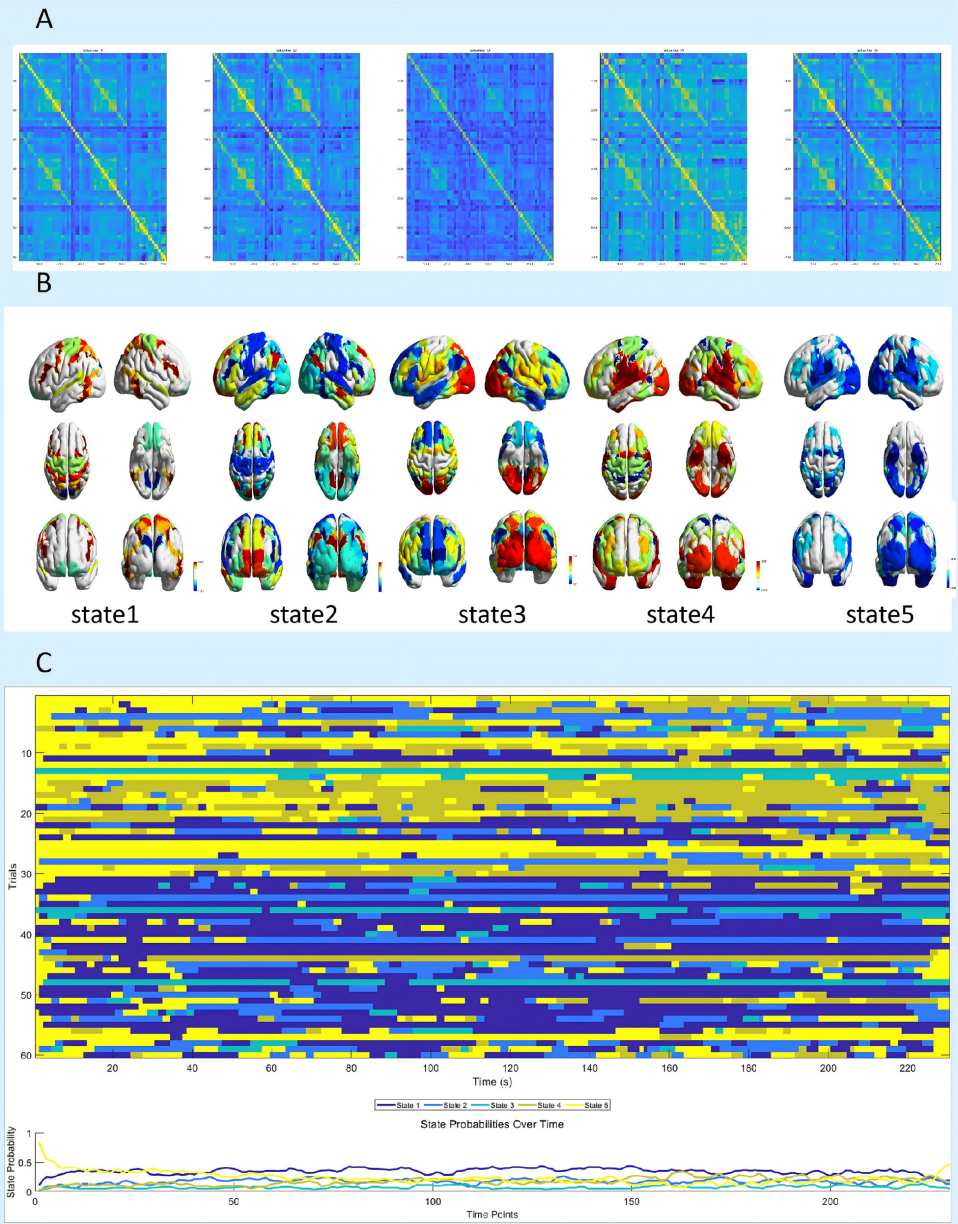
**Characterization of five states. (A)** State‐specific covariance structures: Heatmap representation of covariance matrices for five HMM states. Color intensity encodes the magnitude of covariance values between different ROIs, **(B)** Cortical surface‐projected activation patterns, and **(C)** Temporal dynamics of state transitions: Probability trajectories of five HMM states (State 1‐State 5) over 230 time points.

Region of Interest (ROI) time courses were extracted from preprocessed blood‐oxygen‐level‐dependent (BOLD) signals using a dual‐template approach. Subcortical parcellation was performed according to the Tian_Subcortex_S4_3T_2009cAsym template (Tian et al. 2020), which delineates 54 anatomically distinct subcortical regions. Cortical segmentation was implemented using the Yeo 2011 17‐network cortical parcellation (Yeo2011_17Networks_MNI152_Mask) (Thomas Yeo et al. 2011), partitioning the cerebral cortex into 17 functional networks based on resting‐state functional connectivity patterns. This combinatorial approach yielded 71 ROIs (54 subcortical + 17 cortical networks) per participant. Time courses were truncated to 230 time points (original 240, excluding the initial 10 volumes for signal stabilization), followed by temporal filtering (0.01–0.1 Hz bandpass) and nuisance regression (6 motion parameters + global signal) (Figure 1). Based on prior research (Moretto et al., 2022), we evaluated HMM with 2 to 16 states through iterative calculations. The optimization process of the model revealed that the five‐state configuration consistently achieved the lowest free energy stabilization (Figure 1), indicating its status as the optimal parameterization. Subsequently, covariance matrices for the five states were created. Mean brain region activation maps were then derived for each state, and time‐varying state probabilities were characterized (Figure 2).

### 2.3 Data Analysis

Group differences in state‐specific metrics between narcolepsy patients and healthy controls (HC) were assessed using non‐parametric Mann–Whitney *U* tests across all five HMM‐derived states, with Benjamini–Hochberg false discovery rate (FDR) correction applied to control the false discovery rate. Subsequent analyses focused on the three states (State 1, State 4, and State 5) showing significant group differences. Within the narcolepsy cohort, differences in these states between patients with versus without hallucinations were further examined using Mann–Whitney *U* tests, with effect sizes quantified by Cohen's *d* (*|d|* ≥ 0.2 = small, ≥ 0.5 = moderate, ≥ 0.8 = large). Associations between Epworth Sleepiness Scale (ESS) scores and state metrics were evaluated via Spearman's rank correlation (*ρ*), with correlation strength classified as weak (|*ρ*| < 0.3), moderate (0.3–0.5), or strong (0.5). For hallucination subgroup comparisons and ESS correlations, FDR correction was separately applied to the three states. All analyses were performed in R v4.5.0 using the rstatix, effsize, and ggplot2 packages, with visualizations generated to illustrate key findings.

## 3 Results

### 3.1 State Characterization

**FIGURE 3:**
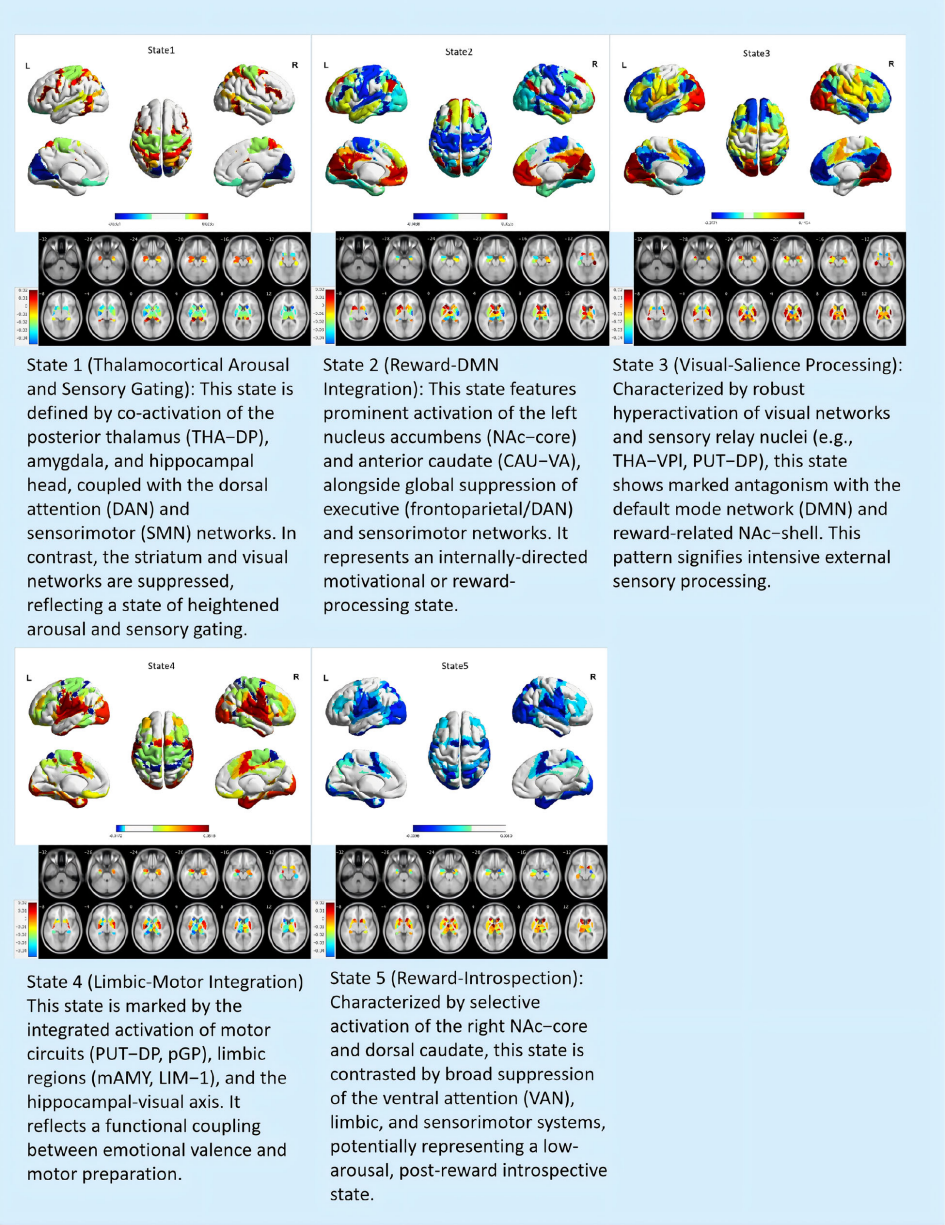
**Activation patterns of HMM‐derived brain states**. Cortical surface‐projected mean activation patterns (upper panels) and corresponding subcortical nuclear activation profiles (lower panels) across five HMM‐identified brain states (States 1–5). Warm colors (red‐yellow) indicate positive activation (*β* > 0), while cool colors (blue) denote suppression (*β* < 0).

The Hidden Markov Model (HMM) analysis identified five distinct dynamic brain states (States 1–5) across both groups, characterized by unique spatial activation patterns. Cortical surface‐projected mean activation profiles and corresponding subcortical nuclear activation patterns for these states are systematically delineated in Figure 3.​

State 1 was defined by thalamocortical arousal and sensory gating, with robust activation of the posterior thalamic nuclei (THA‐DP‐lh': 0.025; THA‐DP‐rh': 0.016), bilateral amygdala (mAMY‐lh': 0.013; mAMY‐rh': 0.011), and hippocampal head (HIP‐head‐m2‐rh': 0.015), accompanied by suppression of striatal subsystems (ventral putamen: PUT‐VP‐lh' −0.014; dorsal caudate: CAU‐DA‐rh' −0.019) and visual networks (VIS‐1: –0.010; VIS‐2: –0.032). This state exhibited strong functional coupling between the dorsal attention network (DAN‐1: 0.020; DAN‐2: 0.015) and the sensorimotor network (SMN‐1: 0.011; SMN‐3: 0.013).

State 2 emerged as a Reward‐DMN integration state, featuring activation of the left nucleus accumbens core (NAc‐core‐lh': 0.032), left ventral anterior caudate (CAU‐VA‐lh': 0.018), and left anterior ventroanterior thalamus (THA‐VAia‐lh': 0.032), alongside global suppression of executive networks (DAN‐2: −0.022; frontoparietal network FP‐3: −0.049; sensorimotor SMN‐1/2/3: −0.034 to −0.038).

State 3 was dominated by visual‐salience detection, with hyperactivation of primary visual networks (VIS‐2: 0.115; VIS‐1: 0.087), bilateral thalamic nuclei (THA‐VPl‐rh': 0.055; THA‐DP‐lh': 0.041), and posterior putamen (PUT‐DP‐rh': 0.052), while default‐mode networks (DMN‐1: −0.077; DMN‐2: −0.051) and bilateral nucleus accumbens shells (NAc‐shell‐lh': −0.057; NAc‐shell‐rh': −0.058) were suppressed.

State 4 exhibited basal ganglia‐limbic‐sensorimotor integration, characterized by enhanced motor circuits (bilateral PUT‐DP: 0.036–0.024; left pGP‐lh': 0.051), limbic activation (mAMY‐lh': 0.037; LIM‐1: 0.046), and visual‐hippocampal coupling (VIS‐1: 0.039; HIP‐head‐l‐rh': 0.030), with suppression of the dorsal attention network (DAN‐1: −0.017) and left anterior thalamus (THA‐VAia‐lh': −0.015).

Finally, State 5 represented a reward‐introspection state, with selective activation of the right NAc‐core (rh': 0.013), bilateral dorsal caudate (CAU‐DA‐rh'/lh': 0.017–0.011), and left THA‐VAia (lh': 0.020), contrasting with global suppression of attentional/limbic systems (VAN‐1: −0.040; mAMY‐rh': −0.048) and sensorimotor/visual networks (SMN‐2: −0.029; VIS‐1: −0.029).

### 3.2 Group Differences in FO

**FIGURE 4:**
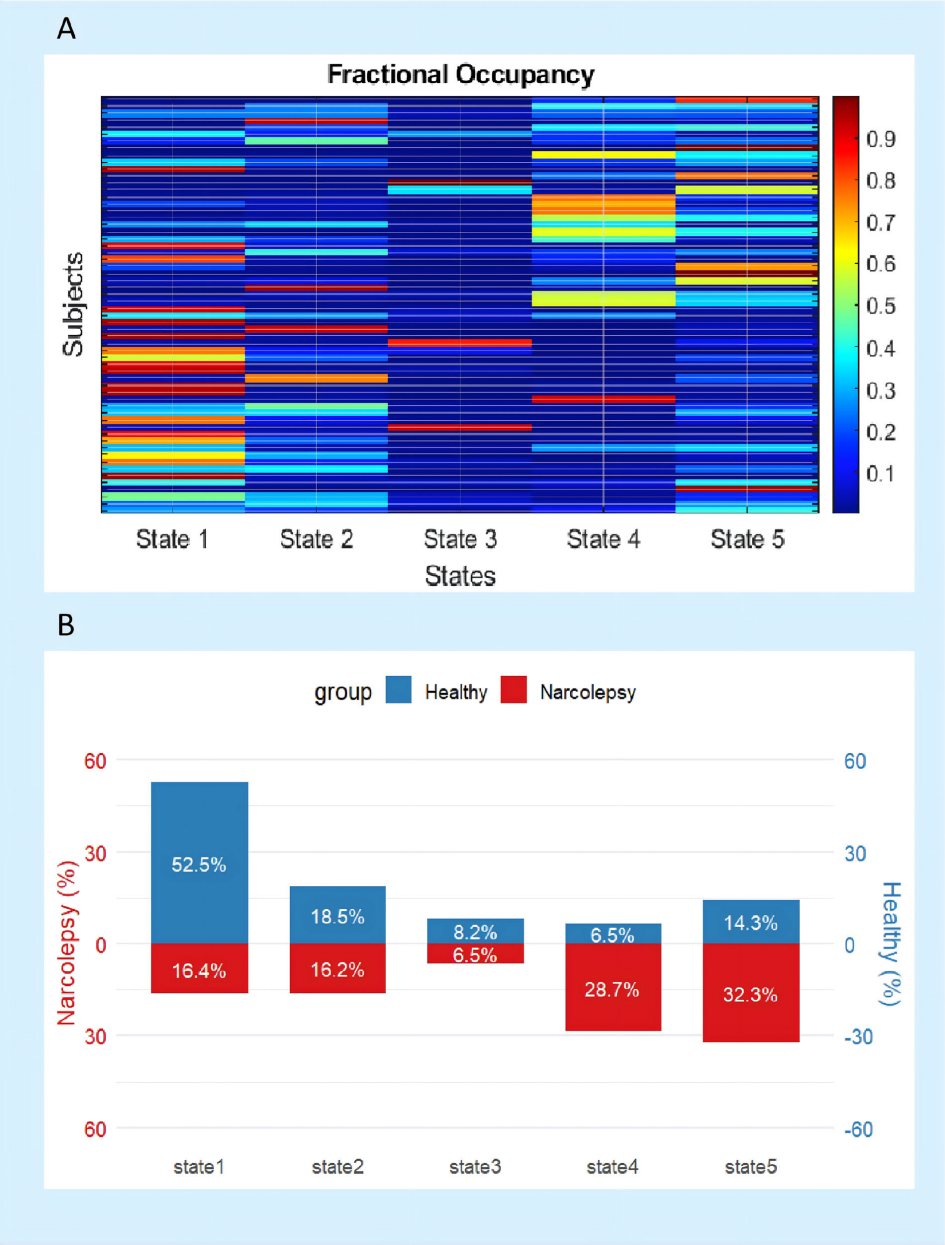
**Fractional occupancy of five states**. (A) Heatmap visualization of state fractional occupancy for individual subjects (rows: first 30 narcolepsy patients, last 30 healthy controls) across five states (columns). Color intensity (jet colormap) indicates the proportion of time spent in each state, scaled from 0% (blue) to 100% (red) and (B) This dual‐axis visualization illustrates the percentage distribution of narcolepsy patients (left axis, red bars) and healthy controls (right axis, blue bars) across five states.

Statistical comparisons using the Mann–Whitney *U* test with FDR‐corrected *p*‐values revealed significant differences in state‐specific probability distributions between the patient and control groups (*n* = 30 per group). Narcolepsy patients exhibited marked reductions in State 1 proportions compared to healthy controls (*d* = −1.16, *p*_FDR_ < 0.001) and pronounced elevations in State 4 (*d* = 1.02, *p*_FDR_ < 0.001). State 5 proportions were also significantly higher in patients (*d* = 0.70, *p*_FDR_ = 0.036). In contrast, State 3 showed a marginal between‐group difference that approached but did not reach statistical significance (*d* = −0.08, *p*_FDR_ = 0.058), while State 2 demonstrated no discernible group‐level variation (*d* = −0.10, *p*_FDR_ = 0.328). These findings highlight robust heterogeneities in States 1, 4, and 5, with clinically meaningful effect sizes (0.8) underscoring their biological relevance, whereas States 2 and 3 showed minimal discriminatory power (as illustrated in Figure 4 and Table 1). Detailed clinical characteristics and state‐specific fractional occupancy (FO) values for all five dynamic states in narcolepsy patients are provided in Supplementary Table S2.​

**TABLE 1:** Comparison of state proportions between groups.

State	Mann–Whitney *U*	*p*‐value	*p*_FDR_	Cohen's *d*	Effect size magnitude	Significance
State 1	156	5.29 × 10^−6^	2.64 × 10^−5^	−1.16	Large	***
State 2	383	0.328	0.328	−0.10	Negligible	ns
State 3	315	0.0462	0.0578	−0.08	Negligible	ns
State 4	727	2.07 × 10^−5^	5.18 × 10^−5^	1.02	Large	***
State 5	605	0.0216	0.036	0.7	Moderate	*

*Note*: Significance thresholds: ***(*p*_FDR_ < 0.001), **(*p*_FDR_ < 0.01), *(*p*_FDR_ < 0.05).

Effect size interpretation: |*d*| ≥0.8 = large, ≥0.5 = moderate, ≥0.2 = small, <0.2 = negligible. FDR correction via Benjamini–Hochberg procedure. Directionality of Cohen's *d*: Negative values indicate higher proportions in Controls, positive values in Patients.

Abbreviation: ns, not significant.

### 3.3 Clinical Correlations

**FIGURE 5:**
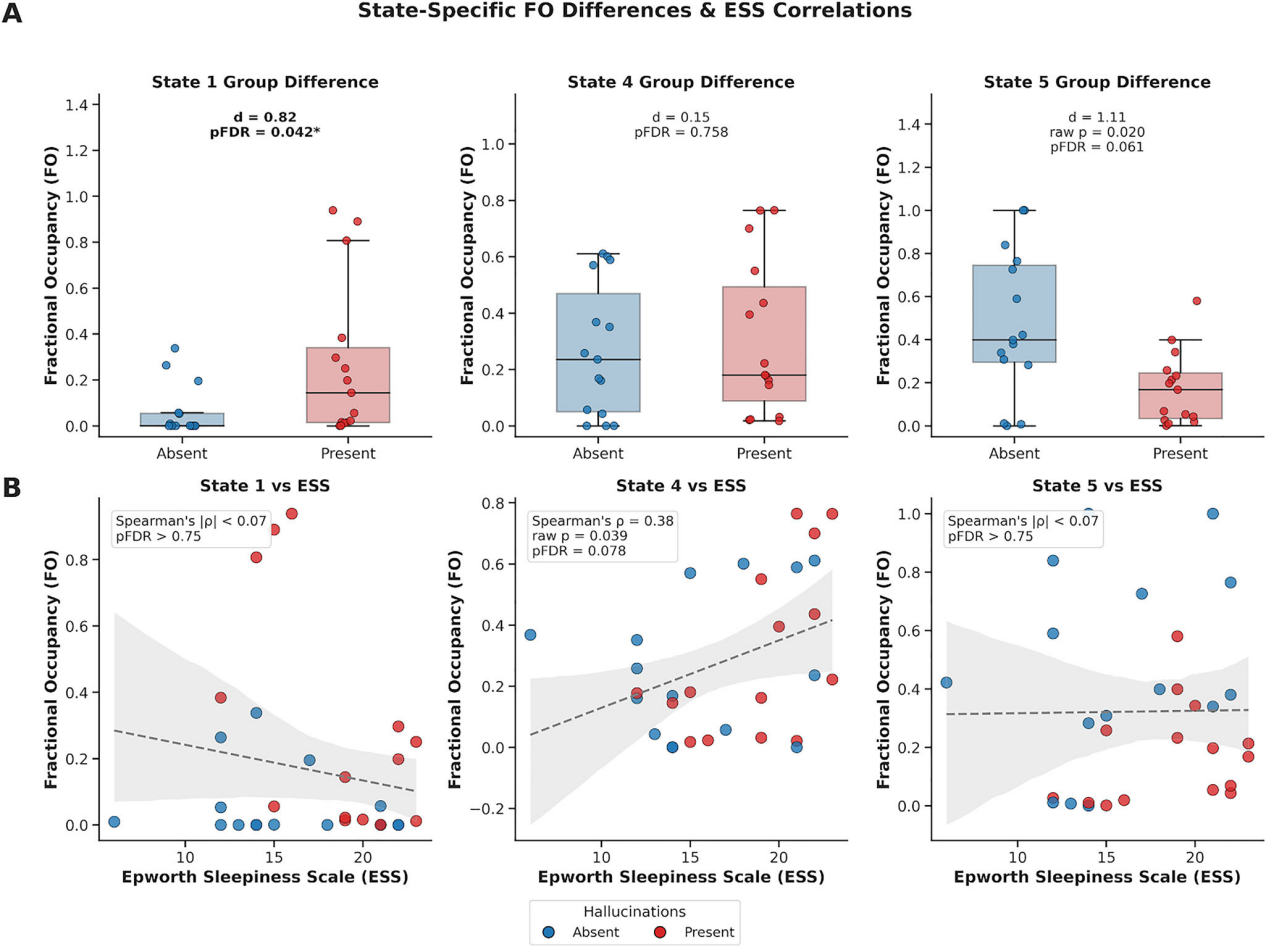
**Associations of state‐specific fractional occupancy (FO) with hallucinations and daytime sleepiness**. (A) FO differences between hallucination‐present (red, n = 15) and ‐absent (blue, *n* = 15) groups. State 1 was significantly increased in the present group (*d* = 0.82, *p*_FDR_ = 0.042), while State 5 showed a trend toward reduction (*d* = 1.11, *p*_FDR_ = 0.061). (B) Correlations between Epworth Sleepiness Scale (ESS) and FO. State 4 exhibited a moderate positive correlation trend with ESS (*ρ* = 0.38, *p*_FDR_ = 0.078). Data were analyzed using Mann–Whitney *U* tests and Spearman correlations with Benjamini–Hochberg FDR correction (m = 6).

Significant differences in state‐specific fractional occupancy (FO) were observed between narcolepsy patients with versus without hallucinations. After Benjamini‐Hochberg false discovery rate (FDR) correction, State 1 FO was significantly higher in the hallucination‐present group compared to the hallucination‐absent group (*d* = 0.82, *p*_FDR_ = 0.042). Notably, State 5 exhibited a nominally significant reduction in the hallucination‐present group (*d* = 1.11, *raw p* = 0.020), though this did not survive FDR correction (*p*_FDR_ = 0.061). No significant association was found between hallucinations and State 4 (*d* = 0.15, *p*_FDR_ = 0.758) (as illustrated in Figure 5 and Table 2).

**TABLE 2:** Associations between clinical features and HMM‐derived state metrics.

Feature	State	Test	*p*‐value	Effect size	Direction	*p*_FDR_	Significance
Hallucinations	State 1	Mann–Whitney *U*	0.007	*d* = 0.82	Higher in present	0.042	*
Hallucinations	State 4	Mann–Whitney *U*	0.709	*d* = 0.15	Higher in present	0.758	ns
Hallucinations	State 5	Mann–Whitney *U*	0.020	*d* = 1.11	Lower in present	0.061	ns
Epworth	State 1	Spearman's *ρ*	0.757	*ρ* = −0.06	Negative	0.758	ns
Epworth	State 4	Spearman's *ρ*	0.039	*ρ* = 0.38	Positive	0.078	ns
Epworth	State 5	Spearman's *ρ*	0.730	*ρ* = 0.07	Positive	0.758	ns

*Note*: Effect size: Cohen's *d* thresholds: |*d*| ≥0.2 (small), ≥0.5 (moderate), ≥0.8 (large); Spearman's *ρ* strength: |*ρ*| <0.3 (weak), 0.3–0.5 (moderate), >0.5 (strong). Significance: *p_FDR_ <0.05; ns = non‐significant. Direction: For hallucinations, “Higher/Lower in Present” indicates median values in hallucination‐present vs. absent groups; for Epworth, “Positive/Negative” denotes correlation direction.

For daytime sleepiness measures, Epworth Sleepiness Scale (ESS) scores showed a moderate positive correlation with State 4 FO (Spearman's *ρ* = 0.38, raw *p* = 0.039) that trended toward significance after correction (*p*_FDR_ = 0.078). Neither State 1 nor State 5 demonstrated meaningful correlations with ESS scores (*|ρ|* < 0.07, *p*_FDR_ > 0.75) (as illustrated in Figure 5 and Table 2).

## 4 Discussion

This study provides the first characterization of dynamic brain state abnormalities in NT1 using HMM. Our analysis identified five distinct brain states that reveal novel pathophysiological mechanisms underlying NT1 symptoms. The tripartite abnormalities observed—comprising thalamocortical destabilization, striatal‐limbic hyperintegration, and reward network disinhibition—establish a novel framework for interpreting NT1's core symptomatology through the lens of dynamic brain state transitions. The identified alterations in FO of specific states, coupled with their clinical correlations, offer a systems‐level perspective on the neuropathological mechanisms underlying narcolepsy symptoms.

Notably, our findings converge with prior work by Drissi et al. (2016), who used simultaneous EEG‐fMRI to report disrupted temporal dynamics of EEG microstate A—a configuration linked to the DMN—in adolescents with NT1. Although their approach captured millisecond‐scale electrophysiological fluctuations while ours reflects second‐scale hemodynamic network interactions, the convergence on DMN‐related dysregulation across these distinct methodologies underscores its central role in narcolepsy pathophysiology. This multi‐scale consistency strengthens the interpretation that DMN instability is a robust hallmark of NT1, manifesting from rapid cortical field configurations to sustained whole‐brain network states.

### 4.1 State 1 (Thalamocortical Arousal and Sensory Gating) and Its Association With Narcolepsy

State 1 exhibited a distinct neurophysiological profile characterized by co‐activation of the thalamus (predominantly posterior nuclei), amygdala, and hippocampal head, concurrent with suppression of striatal subsystems and visual networks (VIS‐1/VIS‐2). This configuration resembles a high‐vigilance brain state in which thalamocortical circuits support wakefulness while filtering out irrelevant sensory input—particularly visual noise. Consistent with orexin deficiency destabilizing arousal networks, NT1 patients as a group exhibited significantly reduced fractional occupancy of State 1, likely contributing to general vigilance instability and susceptibility to sleep attacks. Strikingly, however, patients who reported hallucinations showed elevated State 1 occupancy compared to those without hallucinations. Given that hallucinations in NT1 typically occur during sleep–wake transitions (e.g., hypnagogic or hypnopompic periods), this finding suggests that State 1 may be aberrantly recruited during these boundary states. A plausible interpretation of the elevated State 1 occupancy in patients with hallucinations is that these individuals engage thalamo‐limbic arousal circuits more intensely during sleep‐wake transitions in an effort to sustain wakefulness. In healthy individuals, such activation would support stable alertness. In narcolepsy type 1, however, orexin deficiency is thought to impair thalamic sensory gating. As a result, heightened activity in this state may amplify internally generated signals from limbic and hippocampal regions, while concurrent suppression of primary visual networks reduces reliance on external input. This combination could lead the brain to misattribute endogenous dream‐like imagery as real sensory perceptions, thereby giving rise to hypnagogic or hypnopompic hallucinations. Thus, rather than reflecting intact vigilance, increased State 1 occupancy in this subgroup may signify a maladaptive attempt to maintain arousal at the cost of perceptual fidelity. While this interpretation remains hypothetical and cannot be temporally localized without simultaneous EEG, it offers a testable model: State 1 may represent a “fragile vigilance” state whose maladaptive expression during transitional periods contributes to perceptual distortions in narcolepsy.

### 4.2 State 4 (Basal Ganglia‐Limbic‐Sensorimotor Integration) and Its Positive Correlation With Sleepiness Scores

State 4 was characterized by: Hyperactivation of motor‐limbic circuits, including the putamen, globus pallidus (motor inhibition nodes), and amygdala (limbic arousal), enhanced visual‐hippocampal coupling; and concurrent suppression of the dorsal attention network (DAN)​. This configuration recapitulates core neurophysiological features of REM sleep, including limbic hyperactivity, sensorimotor decoupling, and internally generated visual imagery. In NT1, the loss of orexin‐mediated stabilization may permit such REM‐like states to intrude into wakefulness, disrupting sustained cortical alertness. Notably, fractional occupancy of State 4 showed a nominally positive but non‐significant association after FDR correction with Epworth Sleepiness Scale scores, suggesting that its frequent occurrence contributes to excessive daytime sleepiness. Although we did not assess cataplexy frequency directly, the co‐activation of the amygdala—known to process emotional salience—and basal ganglia output nuclei (globus pallidus) aligns conceptually with the neural substrate of cataplexy, wherein strong positive emotions trigger brainstem‐mediated motor atonia. Thus, State 4 may represent a dissociated REM‐like state potentially preceding motor atonia that bridges emotional arousal and motor inhibition, even in the absence of full cataplectic attacks. The concurrent engagement of visual and hippocampal regions in this state further supports the intrusion of dream‐like mentation into wakefulness, a hallmark of NT1 phenomenology. This observation is consistent with prior static resting‐state findings of increased visual‐hippocampal connectivity in adolescent NT1 patients (Fulong et al. 2020) and abnormal spontaneous activity (fALFF) in limbic and visual cortices (Wu et al. 2022). Moreover, simultaneous EEG‐fMRI studies have demonstrated that dynamic brain configurations closely track vigilance fluctuations and are coupled with slow‐wave activity during sleep–wake transitions (Zhou et al. 2019). While these approaches differ methodologically from our HMM‐based decomposition, they collectively underscore that visual–limbic dysregulation is a reproducible feature of NT1. Our results extend this literature by identifying State 4 as a recurrent, whole‐brain dynamic signature that integrates these abnormalities into a single maladaptive configuration during wakefulness.

### 4.3 State 5 (Reward‐Introspection State) and Its Specificity in Narcolepsy

State 5 showed selective activation of reward circuits (nucleus accumbens, dorsal caudate) but global suppression of attentional/limbic systems and visual networks (VIS‐1↓). This configuration bears resemblance to theoretical models of internally oriented cognition, during which mesolimbic reward circuits are engaged while external sensory input is gated (Dang‐Vu et al., 2010). In narcolepsy type 1, the loss of orexin‐mediated stabilization of arousal states may permit such REM‐like configurations to intrude into wakefulness, even in the absence of overt sleep onset. Critically, while emotional triggers such as laughter or excitement are well‐known precipitants of cataplexy, the neural signature of cataplexy appears more closely aligned with State 4, which integrates limbic (amygdala), motor (putamen/globus pallidus), and visual‐hippocampal activity. In contrast, State 5 lacked prominent motor or amygdalar co‐activation and did not correlate with either hallucinations or Epworth Sleepiness Scale scores in our cohort. This dissociation suggests that elevated State 5 occupancy may reflect a distinct facet of REM‐state dysregulation: one involving reward‐related processing and internally oriented cognition, rather than motor inhibition or perceptual disruption. Thus, State 5 may represent a “quiet” REM‐like intrusion—characterized by dream‐like mentation, altered motivational salience, or emotional tone modulation—without the motor or sensory distortions that define core NT1 symptoms (Thorpy et al. 2024). Although the term “quiet” is used here descriptively to denote the absence of overt behavioral correlates, this state's functional significance remains speculative. Future studies incorporating affective tasks or dream‐enactment assessments could test whether this state contributes to the less‐quantified, yet clinically meaningful, aspects of narcolepsy phenomenology.

### 4.4 Thalamic Gating Dysfunction and Dissociated REM Sleep Phenomena

The hallmark of NT1 is the dissociation of REM sleep components into wakefulness—most notably, cataplexy (sudden loss of muscle tone triggered by emotions), hypnagogic hallucinations, and sleep‐onset REM periods. Our HMM findings provide a dynamic systems‐level account of this pathophysiology. Specifically, reduced occupancy of State 1 (thalamocortical arousal) reflects impaired maintenance of a stable wakeful baseline, thereby lowering the threshold for REM‐related phenomena to intrude into consciousness. Concurrently, elevated expression of State 4—characterized by co‐activation of limbic regions (amygdala), motor control nuclei (putamen, globus pallidus), and visual‐hippocampal circuits, alongside suppression of dorsal attention networks—may directly underlie cataplexy. During emotional arousal, amygdala activation in State 4 could engage brainstem REM atonia pathways via basal ganglia outputs, while the concurrent suppression of cortical motor monitoring (via DAN inhibition) prevents conscious override of muscle atonia. Thus, State 4 may represent a “wakeful REM‐atonia mimic”: a maladaptive brain configuration in which emotional salience triggers subcortical motor inhibition despite preserved environmental awareness. Similarly, increased State 5—marked by reward circuit activation and sensory suppression—may reflect other REM‐like features, such as dream mentation or emotional lability during wakefulness. Together, these findings extend the classic “thalamic filter failure” hypothesis by demonstrating its dynamic network manifestation: orexin deficiency destabilizes thalamocortical gating, leading not only to reduced State 1 (failed signal amplification) but also to pathological overexpression of limbic‐subcortical states (States 4–5) that recapitulate core REM intrusion symptoms, including hallucinations, excessive sleepiness, and critically, cataplexy.

### 4.5 Therapeutic Implications

Although our study is observational and does not evaluate interventions, the identification of state‐specific network abnormalities provides a potential roadmap for future circuit‐targeted therapies in NT1. Specifically, the reduced occupancy of State 1 reflects impaired thalamocortical arousal, suggesting that approaches designed to enhance thalamic excitability or stabilize sensory gating might help alleviate symptoms related to vigilance instability, such as hallucinations or sleep attacks. In contrast, the pathological overexpression of States 4 and 5—both of which resemble REM‐like patterns involving limbic activation and motor inhibition—implicates subcortical‐limbic circuits as candidate targets for modulating cataplexy and excessive daytime sleepiness. Emerging neuromodulation strategies, including transcranial magnetic stimulation applied to thalamocortical nodes, could be explored in preclinical models or early‐phase clinical trials, with HMM‐derived brain states serving as dynamic biomarkers to guide intervention timing and target selection.

### 4.6 Limitations and Future Directions

While our HMM approach overcomes limitations of static connectivity analyses, several considerations warrant attention: The cross‐sectional design precludes causal interpretations of state‐symptom relationships. 8‐min scan durations may underrepresent ultradian state cycling patterns. A key limitation of our study is the absence of direct cerebrospinal fluid (CSF) hypocretin‐1 measurements. While all patients met stringent clinical criteria for NT1 (cataplexy‐positive), individual variability in residual orexin signaling could modulate brain state dynamics.

It should be noted that, unlike Drissi et al. (2016), our study used resting‐state fMRI alone, without concurrent EEG. While simultaneous fMRI‐EEG effectively links electrophysiological and hemodynamic dynamics, fMRI‐based HMM offers unique advantages in characterizing whole‐brain network reconfigurations at the systems level—specifically, identifying recurring, spatially distributed brain states that reflect coordinated activity across large‐scale intrinsic networks, which scalp EEG microstates cannot fully capture. Thus, our fMRI‐only approach provides a complementary perspective, revealing how DMN and thalamocortical dysregulation in narcolepsy manifest as sustained (seconds‐long) whole‐brain network interactions. The convergence of these findings with millisecond‐scale EEG microstate results strengthens—rather than weakens—the conclusion that large‐scale network instability is a robust, multi‐scale hallmark of narcolepsy.

From a mechanistic standpoint, however, the link between orexin loss and our findings is biologically plausible: orexin neurons project densely to thalamic relay nuclei and exert depolarizing effects that promote tonic firing and suppress burst‐mode activity (Kolaj et al. 2007). Without this stabilizing input, thalamocortical circuits become prone to state fluctuations, manifesting as reduced State 1 stability. Thus, the HMM abnormalities we observe likely represent the large‐scale BOLD signature of orexin‐deficient thalamic dysregulation. Future studies combining CSF orexin levels, multimodal imaging, and HMM will be essential to test this causal chain.

## 5 Conclusions

In conclusion, this study provides the first characterization of dynamic brain state alterations in NT1 using hidden Markov modeling. We identify a tripartite pattern of whole‐brain network dysregulation—marked by reduced occupancy of a thalamocortical arousal state (State 1) and elevated expression of two REM‐like configurations involving basal ganglia–limbic–sensorimotor (State 4) and reward–introspection (State 5) networks. Importantly, these state‐specific abnormalities are differentially associated with core clinical features: greater fractional occupancy of State 1 is observed in patients with hallucinations, while State 4 shows a positive association with excessive daytime sleepiness. Although causal inferences cannot be drawn from cross‐sectional data, these findings suggest that dynamic instability in thalamocortical and limbic‐subcortical circuits may contribute to the pathophysiological heterogeneity of NT1. Collectively, our results position HMM‐derived brain states as candidate systems‐level biomarkers that reflect the neural correlates of symptom variation in NT1, offering a framework for future longitudinal and interventional studies.

## Author Contributions

All authors have read and approved the final version of the manuscript. Z. G. contributed to conceptualization, methodology, supervision, project administration, and writing – review and editing. W. L. contributed to conceptualization, methodology, software development, formal analysis, data curation, visualization, writing – original draft, writing – review and editing, and funding acquisition. L. J. contributed to investigation, data curation, and writing – review and editing. X. W. and Y. Z. provided resources and contributed to validation. Z. L. contributed to conceptualization, methodology, investigation, and writing – review and editing.

## Declaration

No AI was used for scientific content generation.

## Funding

This work was supported by the Health Commission of Hebei Province 20241036.

## Conflicts of Interest

The authors declare no conflicts of interest.

## Supporting information




**Supplementary materials**: brb371338‐sup‐0001‐TableS1‐S2.doc

## Data Availability

Data sharing is subject to ethics restrictions and therefore the data will be shared on request addressed to Wenyi Li (wendydoctor@163.com) and subject to a data sharing agreement.
