# Supplementary material for: Dynamic Brain State Alterations in Narcolepsy: A Hidden Markov Model Approach to Thalamocortical Instability and Symptom‐Specific Neural Correlates
Source: Brain Behav. 2026 Mar 31;16(4):e71338. doi: 10.1002/brb3.71338 (PMC13111990; doi:10.1002/brb3.71338)
Supplement: Supplementary file 1 — Supplementary materials: brb371338‐sup‐0001‐TableS1‐S2.doc [file BRB3-16-e71338-s001.doc]

**Supplementary_Table 1. Demographic and clinical characteristics of participants**

| **Variables** | **Narcolepsy group (n=30)** | **Control group (n=30)** | ***p*-value** |
| --- | --- | --- | --- |
| Demographics |  |  |  |
| Age (years) | 27.8 ± 11.4 | 27.7 ± 9.7 | 0.97 |
| Sex (male/female) | 18/12 | 16/14 | 0.61 |
| Clinical symptoms, n (%) |  |  |  |
| Hypnagogic hallucinations | 15 (50.0) | - |  |
| Sleep paralysis | 13 (43.3) | - |  |
| Gustatory changes | 11 (36.7) | - |  |
| Weight fluctuation | 12 (40.0) | - |  |
| Neuropsychological tests |  | - |  |
| Epworth Sleepiness Scale | 17.13 ± 4.3 | - |  |
| HAMD depression score | 6.2 ± 8.7 | - |  |
| HAMA anxiety score | 7.4 ± 12.0 | - |  |
| MMSE total score | 28.23 ± 1.7 | - |  |

Note: Data presented as mean ± SD or n (%).

HAMD = Hamilton Depression Rating Scale; HAMA = Hamilton Anxiety Rating Scale;

MMSE = Mini-Mental State Examination. Dash (-) indicates not applicable.

**Supplementary_Table 2. Clinical characteristics and fractional occupancy (FO) values across 5 states in narcolepsy patients**

| **ID** | **HH** | **SP** | **EDS** | **SOREMPs** | **state1** | **state2** | **state3** | **state4** | **state5** |
| --- | --- | --- | --- | --- | --- | --- | --- | --- | --- |
| NP01 | 2 | 2 | 12 | 4 | 1.46835E-05 | 0.000189224 | 1.31334E-06 | 0.160593486 | 0.839201293 |
| NP02 | 1 | 1 | 20 | 4 | 0.016136277 | 0.246585301 | 1.9101E-06 | 0.394888366 | 0.342388146 |
| NP03 | 1 | 1 | 23 | 4 | 0.250485869 | 0.25606128 | 0.058522253 | 0.221948155 | 0.212982443 |
| NP04 | 1 | 1 | 21 | 3 | 4.20364E-05 | 0.925283845 | 1.91517E-07 | 0.020977627 | 0.0536963 |
| NP05 | 2 | 1 | 6 | 5 | 0.009411654 | 0.20009739 | 0.00068994 | 0.368030996 | 0.42177002 |
| NP06 | 1 | 2 | 12 | 3 | 0.383675304 | 0.129854593 | 0.283218052 | 0.177163629 | 0.026088422 |
| NP07 | 1 | 2 | 19 | 5 | 0.144171947 | 0.461775159 | 5.64732E-05 | 0.1616568 | 0.232339621 |
| NP08 | 2 | 2 | 21 | 2 | 1.39639E-06 | 4.74403E-05 | 1.78766E-07 | 4.58742E-05 | 0.99990511 |
| NP09 | 2 | 2 | 22 | 2 | 0.000166862 | 0.00902017 | 6.12773E-07 | 0.61105412 | 0.379758235 |
| NP10 | 2 | 2 | 14 | 4 | 0.337820345 | 0.202913862 | 0.008561583 | 0.168216948 | 0.282487262 |
| NP11 | 1 | 1 | 16 | 4 | 0.938557 | 0.01978516 | 2.43788E-05 | 0.022718488 | 0.018914974 |
| NP12 | 2 | 2 | 22 | 4 | 2.01786E-05 | 7.90024E-05 | 1.26148E-06 | 0.235685372 | 0.764214185 |
| NP13 | 2 | 1 | 14 | 2 | 1.91418E-05 | 0.003202079 | 0.996763174 | 1.14796E-06 | 1.4457E-05 |
| NP14 | 1 | 1 | 19 | 3 | 0.013630336 | 0.018395986 | 0.356620658 | 0.031286833 | 0.580066187 |
| NP15 | 1 | 1 | 23 | 3 | 0.011794313 | 0.03492065 | 0.021420328 | 0.763970982 | 0.167893726 |
| NP16 | 1 | 1 | 22 | 4 | 0.198159488 | 0.058677933 | 3.36296E-06 | 0.700057127 | 0.04310209 |
| NP17 | 1 | 1 | 21 | 5 | 0.000485302 | 0.037911951 | 5.05714E-07 | 0.764495448 | 0.197106793 |
| NP18 | 1 | 1 | 19 | 3 | 0.021932008 | 0.029234806 | 4.32037E-06 | 0.550131044 | 0.39869782 |
| NP19 | 2 | 1 | 12 | 3 | 0.263790668 | 0.342893735 | 0.031466435 | 0.350982227 | 0.010866935 |
| NP20 | 2 | 1 | 18 | 3 | 1.78957E-05 | 0.000526482 | 4.44207E-06 | 0.600793575 | 0.398657605 |
| NP21 | 1 | 2 | 22 | 5 | 0.296951258 | 0.179607873 | 0.019374955 | 0.435900855 | 0.06816506 |
| NP22 | 1 | 2 | 15 | 4 | 0.890296374 | 0.0697517 | 0.021736069 | 0.017343164 | 0.000872693 |
| NP23 | 1 | 2 | 15 | 5 | 0.055854712 | 0.417028553 | 0.088945637 | 0.180332739 | 0.257838358 |
| NP24 | 1 | 2 | 14 | 4 | 0.806898051 | 0.038155108 | 1.34609E-06 | 0.145101014 | 0.009844481 |
| NP25 | 2 | 2 | 17 | 3 | 0.195282886 | 0.02194751 | 1.10591E-06 | 0.057053144 | 0.725715354 |
| NP26 | 2 | 2 | 14 | 5 | 1.42676E-05 | 1.64062E-05 | 2.37094E-07 | 0.000224716 | 0.999744373 |
| NP27 | 2 | 2 | 12 | 5 | 0.052952955 | 0.099345776 | 7.01744E-05 | 0.257946789 | 0.589684305 |
| NP28 | 2 | 2 | 13 | 3 | 2.40482E-05 | 0.949783434 | 2.68068E-07 | 0.043018255 | 0.007173995 |
| NP29 | 2 | 2 | 15 | 5 | 0.000841466 | 0.064343279 | 0.056813517 | 0.570036579 | 0.30796516 |
| NP30 | 2 | 2 | 21 | 4 | 0.056854093 | 0.014871442 | 9.11736E-06 | 0.58885732 | 0.339408029 |

Note:

- Abbreviations: SP, sleep paralysis; HH, hypnagogic hallucinations; EDS, Epworth Sleepiness Scale; SOREMPs, sleep-onset REM periods.

- Binary variables (SP, HH): 1 = Present, 2 = Absent.

- EDS: Total score (range 0–24, higher values indicate greater sleepiness).

- SOREMPs: Number of episodes recorded per night.
